# Supplementary material for: Inducible, virus-free direct lineage reprogramming enhances scalable generation of human inner ear hair cell-like cells
Source: bioRxiv. 2025 Feb 25:2025.02.20.639352. Preprint. [Version 1] doi: 10.1101/2025.02.20.639352 (PMC11888184; doi:10.1101/2025.02.20.639352)
Supplement: Supplement 1 [file NIHPP2025.02.20.639352v1-supplement-1.pdf]

bioRxiv preprint doi: https://doi.org/10.1101/2025.02.20.639352; this version posted February 25, 2025. The copyright holder for this preprint (which was not certified by peer review) is the author/funder, who has granted bioRxiv a license to display the preprint in perpetuity. It is made available under aCC-BY 4.0 International license.

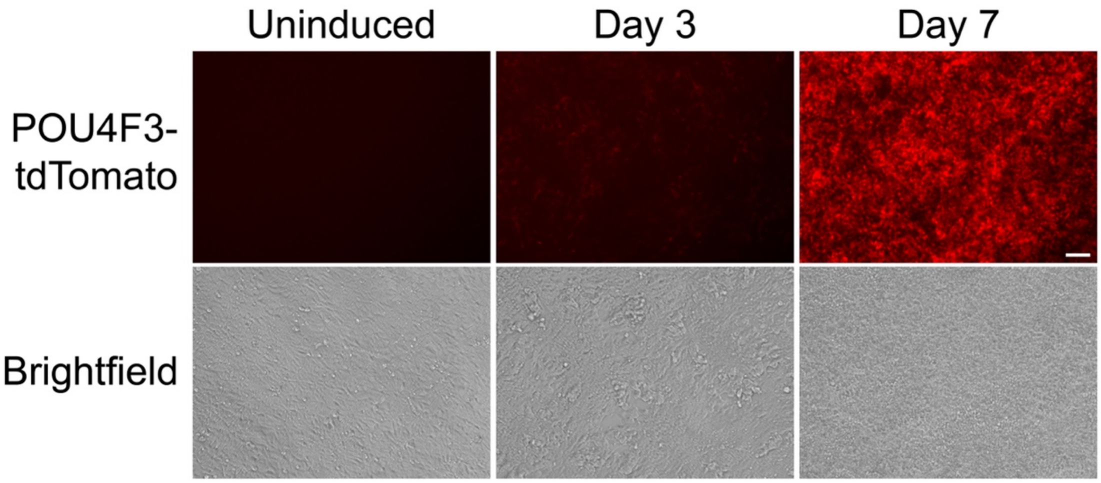

Supplementary Figure 1. The *POU4F3*-tdTomato hair cell reporter becomes visible after ~3 days of reprogramming. Representative images were acquired at 0-, 3- or 7-days post-doxycycline treatment. Scale bar represents 200 µm.

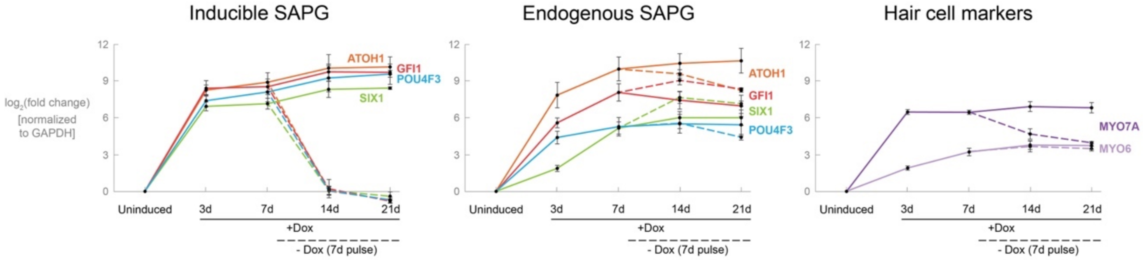

Supplementary Figure 2. RT-qPCR analysis of the cell line treated with continuous doxycycline or a 7-day pulse of doxycycline over the indicated time points. Dashed lines indicate post-doxycycline removal. Values are normalized to GAPDH, and a ratio is calculated by dividing the

bioRxiv preprint doi: https://doi.org/10.1101/2025.02.20.639352; this version posted February 25, 2025. The copyright holder for this preprint (which was not certified by peer review) is the author/funder, who has granted bioRxiv a license to display the preprint in perpetuity. It is made available under aCC-BY 4.0 International license.

uninduced control (0 hour). Fold-change values are log_2-transformed. Error bars indicate SEM. *n* = 3 biological replicates.

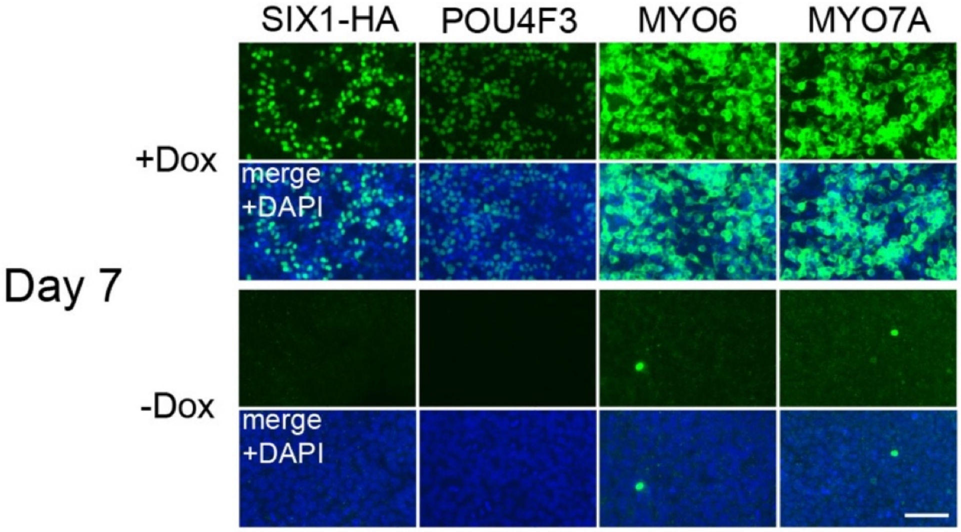

Supplementary Figure 3. Representative pictures of cultures treated with continuous doxycycline for 7 days (+Dox) or left untreated (-Dox).

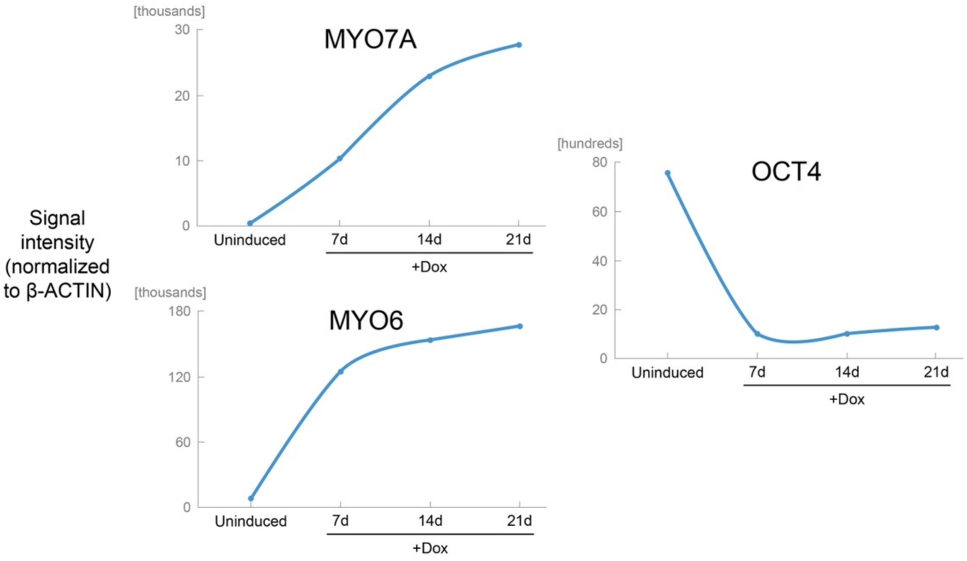

bioRxiv preprint doi: https://doi.org/10.1101/2025.02.20.639352; this version posted February 25, 2025. The copyright holder for this preprint (which was not certified by peer review) is the author/funder, who has granted bioRxiv a license to display the preprint in perpetuity. It is made available under aCC-BY 4.0 International license.

Supplementary Figure 4. Western blot analysis of uninduced control and cells treated with continuous doxycycline for 7, 14, or 21 days. Protein abundance was quantified by normalizing the signal intensity of each target protein to β-ACTIN.

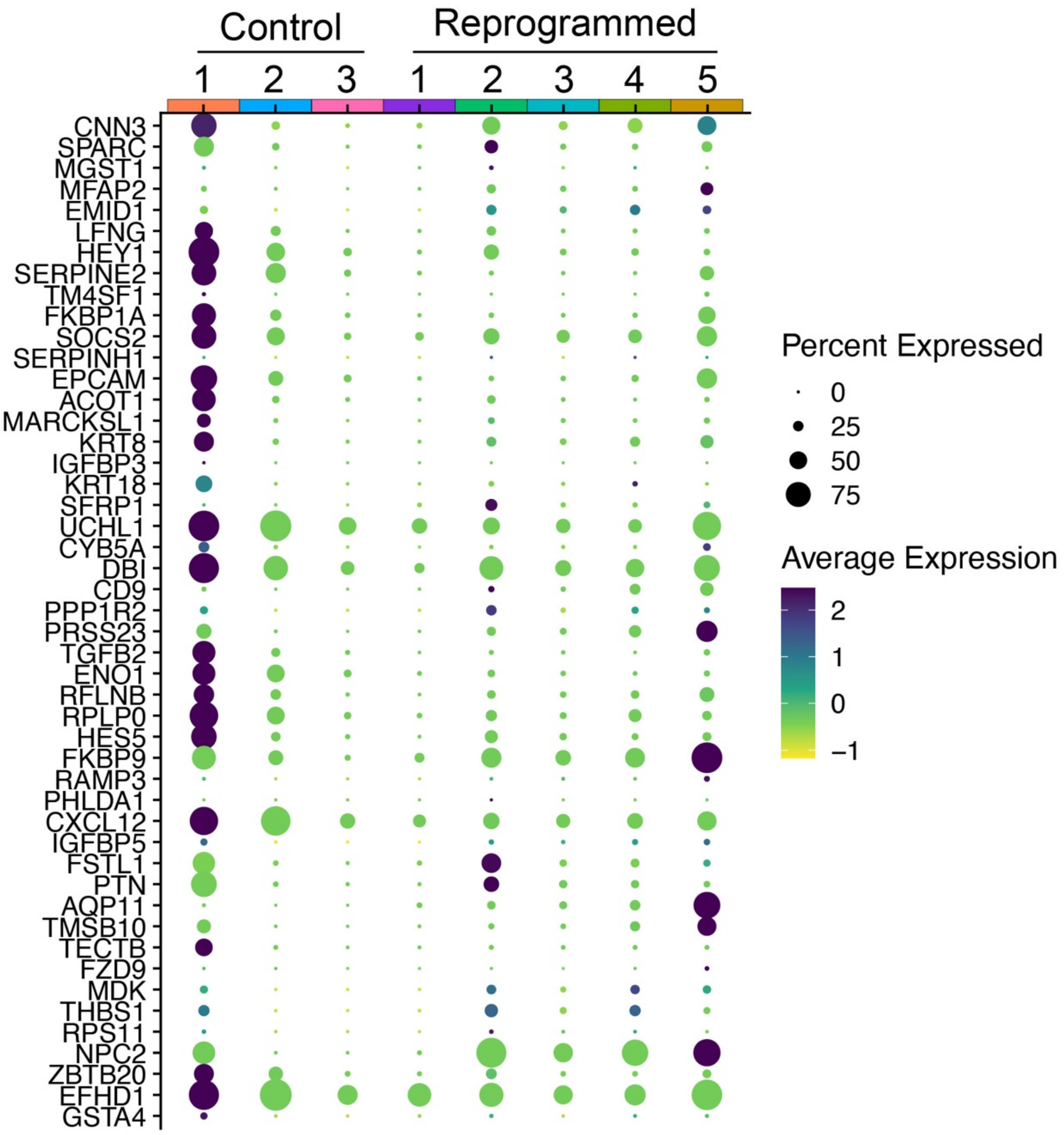

Supplementary Figure 5. Dot plot of the top 50 DE genes in P1 supporting cells as identified by Kolla et al. (2020) in our control and reprogrammed clusters.

bioRxiv preprint doi: https://doi.org/10.1101/2025.02.20.639352; this version posted February 25, 2025. The copyright holder for this preprint (which was not certified by peer review) is the author/funder, who has granted bioRxiv a license to display the preprint in perpetuity. It is made available under aCC-BY 4.0 International license.

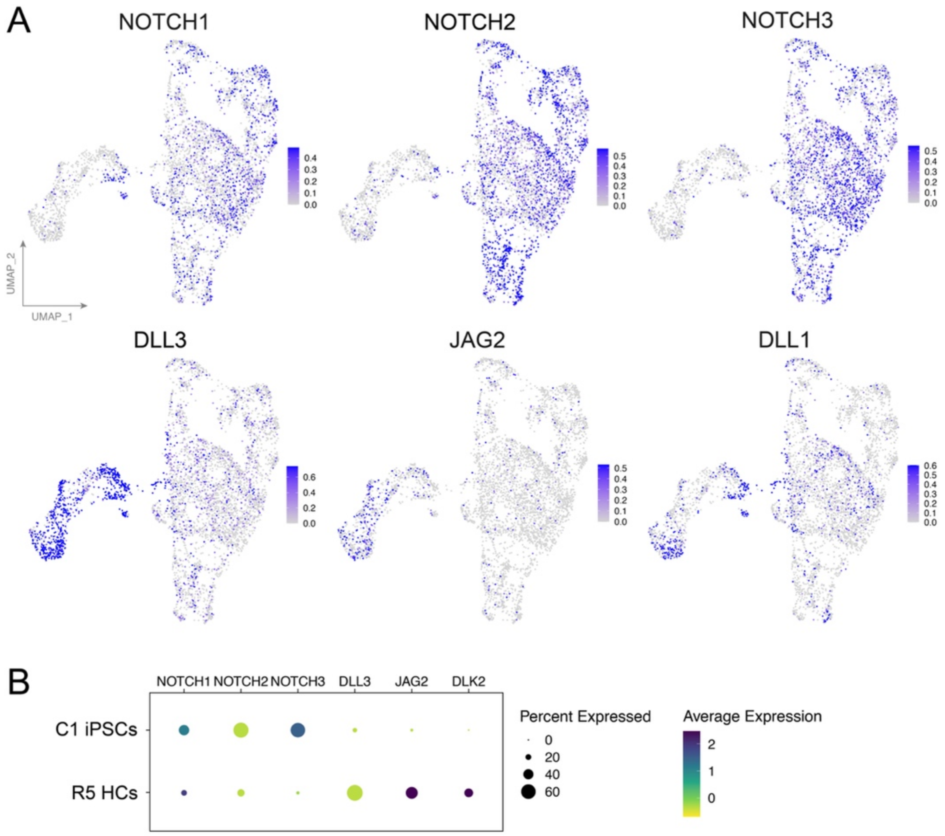

Supplementary Figure 6. HC-like clusters show reduced expression of *NOTCH1/2/3* and increased expression of hair cell-specific NOTCH ligand genes *DLL3*, *JAG2*, and *DLK2* relative to control clusters. (*A*) UMAP projection of Day 21 reprogrammed RV-R1–3 HCs and residual fibroblasts. (B) Dot plot of Day 21 reprogrammed R5 HCs and C1 iPSCs.

bioRxiv preprint doi: https://doi.org/10.1101/2025.02.20.639352; this version posted February 25, 2025. The copyright holder for this preprint (which was not certified by peer review) is the author/funder, who has granted bioRxiv a license to display the preprint in perpetuity. It is made available under aCC-BY 4.0 International license.

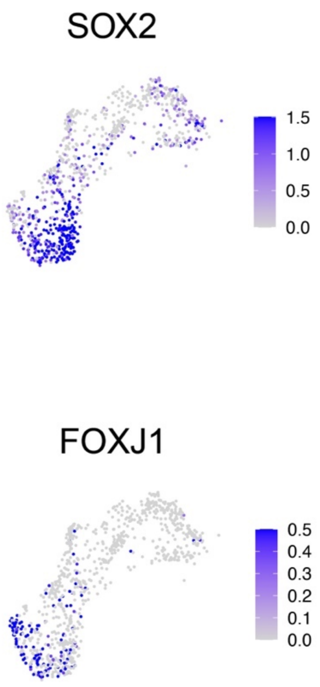

Supplementary Figure 11. RV-R3 HCs shows expression of vestibular hair cell-specific genes *SOX2* and *FOXJ1*. SOX2, required for maintaining Type II vestibular HC identity, marks HC progenitors of both cochlear and vestibular nature but is absent from neonatal cochlear HCs. FOXJ1, involved in ciliogenesis, is expressed in vestibular HCs and neonatal cochlear HCs but not mature cochlear HCs.

bioRxiv preprint doi: https://doi.org/10.1101/2025.02.20.639352; this version posted February 25, 2025. The copyright holder for this preprint (which was not certified by peer review) is the author/funder, who has granted bioRxiv a license to display the preprint in perpetuity. It is made available under aCC-BY 4.0 International license.

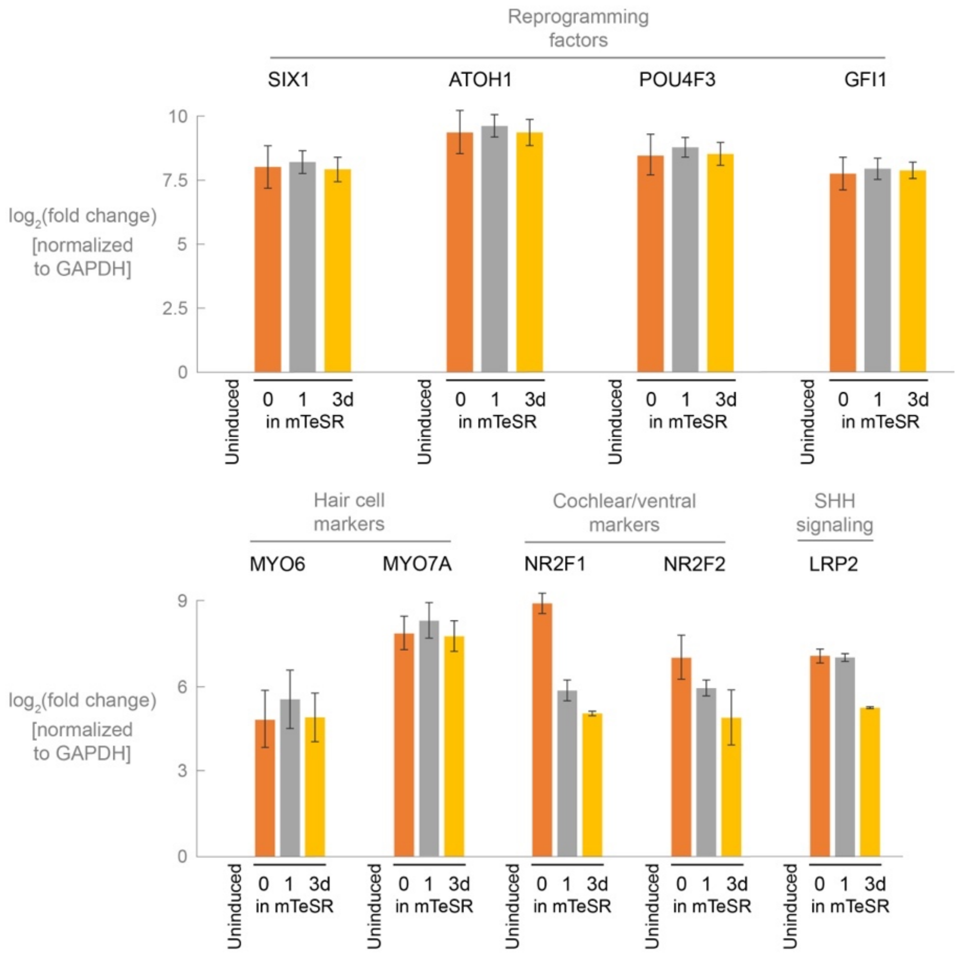

Supplementary Figure 12. RT-qPCR analysis of the cell line treated with continuous doxycycline for 7 days. Cells were treated with doxycycline in mTeSR for 0, 1, or 3 days and moved to hair cell media + doxycycline for the balance of the 7-day reprogramming. Values are normalized to GAPDH, and a ratio is calculated by dividing the uninduced control (0 hour). Fold-change values are log$_2$-transformed. Error bars indicate SEM.

bioRxiv preprint doi: https://doi.org/10.1101/2025.02.20.639352; this version posted February 25, 2025. The copyright holder for this preprint (which was not certified by peer review) is the author/funder, who has granted bioRxiv a license to display the preprint in perpetuity. It is made available under aCC-BY 4.0 International license.

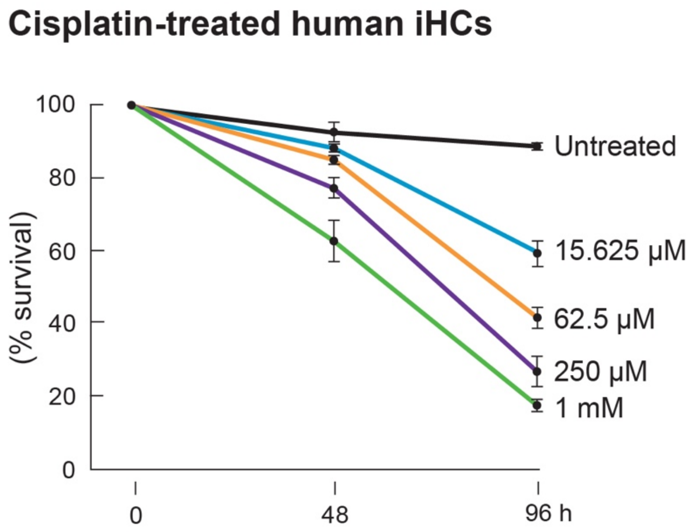

Supplementary Figure 13. Human induced hair cells (iHCs) are sensitive to cisplatin in a dose-dependent manner. Cisplatin ototoxicity profile in human iHCs. Error bars indicate SEM.
